# Die Klimakrise als ethische Herausforderung

**DOI:** 10.1007/s10354-022-00986-3

**Published:** 2022-11-29

**Authors:** Lukas Kenner, Samuel Kenner, Barbara Prainsack, Peter Wallner, Kathrin Lemmerer, Lisbeth Weitensfelder, Hans-Peter Hutter

**Affiliations:** 1 Department of Pathology, Medical University of Vienna, Wien, Österreich; 2 Unit of Pathology of Laboratory Animals, University of Veterinary Medicine Vienna, Wien, Österreich; 3 Bioethics Commission to the Federal Chancellor, Wien, Österreich; 4 Department of Political Science, University of Vienna, Wien, Österreich; 5 Department of Environmental Health, Medical University of Vienna, Wien, Österreich; 6 International Society of Doctors for the Environment, Section Austria, Wien, Österreich

**Keywords:** Ethische Verantwortung, Klimakrise, Gesundheit, Ungerechtigkeit, Ethical responsibility, Climate crisis, Health, Injustice

## Abstract

Der Klimawandel ist eine Gefahr für Gesundheit und soziale Sicherheit von Milliarden von Menschen. Gesundheit und Lebensqualität werden unter dem Einfluss der Klimakrise zunehmend auf vielfältige Weise beeinträchtigt. Der Anstieg der globalen Temperaturen hat häufigere, stärkere Extremwetterereignisse zur Folge, die insgesamt und speziell in der Gesundheitsversorgung zur weiteren Aggravierung von Ungleichheit, Diskriminierung und Ungerechtigkeit beitragen. Zudem begünstigen die klimatischen Bedingungen die Übertragung zahlreicher Infektionskrankheiten und ihr Vordringen in neue Regionen. Sozio-ökonomisch benachteiligte Gebiete mit schwacher Gesundheitsinfrastruktur, etwa im globalen Süden, werden am wenigsten in der Lage sein, ohne gezielte Unterstützung die Folgen der Klimakrise zu bewältigen. Oberstes Gebot ist es, die Verringerung der Treibhausgasemissionen in den Bereichen Verkehr, Energie- und Nahrungsmittelproduktion zu erreichen – auf globaler, nationaler und regionaler Ebene, um negative Gesundheitsfolgen zu mildern. Das Pariser Übereinkommen (2015) ist daher auch als entscheidendes Gesundheitsabkommen zu begreifen. Der Fokus dieses Beitrages liegt auf ethischen Aspekten des Klimawandels im Gesundheitsbereich.

## Abstract (en)

Climate change is a threat to health and social security of billions of people. Health and quality of life are increasingly affected in many ways due to the climate crisis. Rising global temperatures are resulting in increasingly frequent and severe extreme weather events, contributing to further increase in inequality, discrimination, and injustice overall and in health care specifically. Furthermore, climatic conditions are also becoming increasingly suitable for the transmission of infectious diseases and their spread into new regions. Socio-economically disadvantaged regions with weak health infrastructure (e.g. Global South) will be hardly able to cope without specific support. The overriding imperative is to achieve reductions in greenhouse gas emissions from transport, energy and food production at global, national and regional levels to mitigate negative health impacts. The 2015 Paris Agreement must also be seen as crucial health agreement. Our paper aims to highlight ethical aspects of climate change in the health sector.

## Hintergrund

Die Klimakrise bedroht die Grundbedürfnisse des Lebens – Unterkunft, Nahrung und Wasser – und gilt als die größte globale Gefahr für die Gesundheit im einundzwanzigsten Jahrhundert. Sie ist eine Krise an der Schnittstelle Mensch und Umwelt und eines der herausforderndsten Ereignisse dieses Jahrhunderts, das sowohl unser tägliches Leben, unsere Lebensqualität, den sozialen Frieden als auch die geopolitische Weltordnung betrifft. Ihre Auswirkungen bedrohen die Welt als Heimat der Menschen sowie ein friedliches, auf das Wohl aller Menschen gerichtetes Zusammenleben und sie aggravieren bestehende Ungerechtigkeiten. Die immer extremer werdenden Wetterereignisse der letzten Jahre sind nur ein Aspekt dieser Krise, sie sind ein spürbares Zeichen einer allgemein aus den Fugen geratenen Welt. Die Klimakrise verschärft andere soziale Brennherde wie etwa Migration, Konflikte um Ressourcen sowie die Schere zwischen Arm und Reich. Ethische Betrachtungen können uns dabei helfen, von theoretischen Überlegungen und Fakten zum dringend notwendigen Handeln zu kommen. Sich mit dem Klimawandel zu befassen, um ernsthafte und wirksame Maßnahmen für dessen Bewältigung ergreifen zu können, ist nicht nur aus ökologischer Sicht unumgänglich, sondern ebenso sehr ein unabdingbares ethisches und letztlich ärztliches Postulat. Die Klimakrise „provoziert“ ein aus den Werten der Weltgemeinschaft abzuleitendes Handlungsgebot. Die vorliegende Arbeit diskutiert dabei die Herausforderungen, die der Klimawandel an die Ärzteschaft stellt, anhand erfahrungsbasierter Intuition in unterschiedlichen inhaltlichen Kategorien.

## Fünf Herausforderungen für ethisches Handeln

Der Klimawandel und die damit einhergehenden drastischen (gesundheitlichen) Folgen sind eine globale und generationenübergreifende Herausforderung. Die anthropogene Umweltzerstörung, speziell die Gefährdung des Klimas und der biologischen Vielfalt, ist seit langem Realität. Dies bedingt einen moralischen und politischen Auftrag [1] für jene Menschen, die stark zum Klimawandel beitragen und den Großteil der Last ihrer Aktivitäten auf ungerechte Weise auf Menschen in anderen Teilen der Welt und auf zukünftige Generationen abwälzen. Die Klimakrise bedroht alle Menschenrechte [2], insbesondere aber das Recht auf Leben, Freiheit, Eigentum und Sicherheit. Diese basieren auf dem von nahezu allen Kulturen geteilten Wert der Würde des Menschen und dem Anspruch auf ein gutes Leben – in Frieden, Stabilität und in Freiheit von Bedrohungen und Krankheit [3]. Im Folgenden werden fünf wesentliche ethische Herausforderungen im Zuge der Klimakrise, die sich gegenseitig beeinflussen und teils verstärken, genauer herausgearbeitet. Dabei wird als Ausgangspunkt bei allgemeinen menschlichen Bedürfnissen begonnen, bevor auf spezielle Gruppen näher eingegangen wird und letztlich ein übergreifender Fokus auf die nicht-menschliche Umwelt gerichtet wird.

Die *erste ethische Herausforderung* betrifft fundamentale Bedürfnisse und Rechte von Menschen, welche durch den Klimawandel gefährdet sind (Recht auf Leben, Gesundheit, Sicherheit). Für die Ärzteschaft, deren zu gelobender Eid damit beginnt, ihr Leben in den Dienst der Menschlichkeit zu stellen, ist eine Gefährdung dieser Rechte daher auch ein ethisches Anliegen. Die Gesundheit sowie die soziale Sicherheit von Milliarden von Menschen werden unter dem wetterbedingten Einfluss der Klimakrise zunehmend beeinträchtigt. Wenn sich Katastrophen, wie z. B. die Überschwemmungen in Nordrhein-Westfalen im Sommer 2021 oder in Pakistan im Sommer 2022, ereignen, so hat dies zahlreiche unmittelbare Auswirkungen auf das Leben der betroffenen Bevölkerung, die sich in Todesfällen, Verletzungen, einem Anstieg psychischer Erkrankungen sowie materiellen Schäden niederschlagen. Häufig viel zu wenig beachtet werden dabei physische und psychische Langzeitfolgen (Stichwort Posttraumatische Belastungsstörungen, Solastalgie). Verletzt wird zudem auch das Recht auf ökologisches Gleichgewicht, welches als grundlegendes Menschenrecht für das Wohlergehen heutiger und zukünftiger Generationen 1972 auf der Konferenz der Vereinten Nationen über die menschliche Umwelt rechtliche Anerkennung erhielt und in vielen staatlichen Verfassungen der Welt festgeschrieben wurde [3].

Die Weltgesundheitsorganisation (WHO) geht davon aus, dass zwischen 2030 und 2050 pro Jahr etwa 250.000 Menschen an den Folgen des Klimawandels sterben könnten [4] – etwa durch Hitzebelastung. Die weitreichenden Auswirkungen des Klimawandels auf die Gesundheit betreffen auch das Thema Infektionskrankheiten. Die klimatischen Bedingungen werden immer geeigneter für die Übertragung zahlreicher Infektionskrankheiten (z. B. Malaria, Leptospirose), indem sie die biologischen Merkmale von Krankheitserregern (z. B. Wachstum, Überleben, Virulenz) und ihren Vektoren direkt beeinflussen und die Übertragung indirekt durch die Veränderung von Ökosystemen und das veränderte Verhalten der Menschen begünstigen.

Durch die zunehmend auftretenden Extremwetterereignisse drohen auch im Gesundheitsbereich verstärkte Ungleichheit, Diskriminierung und Ungerechtigkeit. Steigende Temperaturen in Kombination mit intensiven Niederschlägen können die Ausbreitung zahlreicher Infektionskrankheiten begünstigen, angefangen bei Vektor übertragenen Krankheiten (z. B. Leishmaniose, Denguefieber) über Infektionen des Magen-Darmtraktes mit Erbrechen und Durchfall (z. B. Cholera, Rotavirus-Infektionen) bis hin zu durch Parasiten ausgelöste Krankheiten wie etwa Bilharziose [5]. Neben vielen körperlichen Gesundheitsrisiken durch extremwetterbedingte Überflutungen sind auch die Auswirkungen auf die psychosoziale Gesundheit nicht zu unterschätzen, wobei bereits vulnerable Gruppen nochmals stärker betroffen sind [6].

Die massiven globalen Folgen der COVID-19-Pandemie für die Gesundheitssysteme und die Volkswirtschaften sollten ein Alarmsignal für politische Entscheidungsträger und auch die Bevölkerung sein, die deutlich angestiegene globale Bedrohungssituation durch bereits jetzt wirksame klimabedingte Veränderungen ernst zu nehmen.

Die Klimakrise wird darüber hinaus erhebliche Auswirkungen haben auf (1) die Kalorienverfügbarkeit pro Kopf, (2) die Unterernährung und Unterentwicklung von Kindern sowie (3) durch Unterernährung bedingte Todesfälle speziell bei Kindern und den massiven Verlust an gesunden Lebensjahren (DALY = Disease Adjusted Live Years) vor allem in Ländern des globalen Südens [7]. Die Ernährungssituation unterliegt seit einigen Jahrzehnten einem rasanten Wandel, welcher in Zusammenhang mit der Klimakrise und ihren Folgen steht: Im Jahr 2000 gab es weltweit etwa gleich viele Menschen, die unter Hunger bzw. an Übergewicht litten – jeweils eine Milliarde [8]. Im Jahr 2009 waren global weiterhin etwa eine Milliarde Menschen unterernährt [9], die Zahl Übergewichtige war jedoch auf zwei Milliarden angestiegen. Auch extremes Übergewicht verzeichnete eine starke Zunahme, so zeigte sich 2016 eine beinahe dreimal so hohe Adipositas-Prävalenz wie im Jahr 1975 [10]. Der Grund liegt darin, dass auch in Schwellenländern immer mehr tierische Produkte statt auf Stärke basierte Lebensmittel konsumiert werden. Der Verzehr von Fleisch hat weltweit stark zugenommen [11]. Dieser Trends hat erhebliche, teils drastische Folgen für die Gesundheit und die Umwelt. Ernährung mit einem hohen Anteil an rotem und verarbeitetem Fleisch führt zu einem erhöhten Risiko für Adipositas und deren Folgen wie z. B. Typ-2-Diabetes, Herz-Kreislauf-Erkrankungen und Krebs (v. a. Karzinome des Gastrointestinaltrakts) und Anstieg der Gesamtmortalität [12–16]. Fleischverzehr hat aber nicht nur negative gesundheitliche Auswirkungen, sondern ebenso auf die globale Landnutzung: Viehhaltung einschließlich der dafür notwendigen Futteranbauflächen nehmen den Großteil der landwirtschaftlichen Flächen in Anspruch und spielen eine große Rolle bei der Abholzung von Flächen [17]. Klimawandelbedingte Veränderungen im Sektor Landwirtschaft sowie Gesundheit stehen somit zeitlich und örtlich in Wechselwirkung und beeinflussen sich gegenseitig durch negative synergistische Auswirkungen. Während die Auswirkungen des Klimawandels diverse Fehl- und Mangelernährungen über den Faktor Landwirtschaft begünstigen, kann die Landwirtschaft selbst wieder deutlich negativ auf den Klimawandel zurückwirken: Das besonders potente Treibhausgas (THG) Methan wird von Rindern ausgestoßen und macht durch den enormen und weiter steigenden Fleischverzehr einen zunehmend größeren Anteil der THG-Emissionen aus [18]. Die mit Methan zusammenhängenden Umweltschäden, aber auch mit dem Verzehr von Fleisch in Verbindung gebrachten Krankheitsbilder wie Fettleibigkeit, Herz-Kreislauf-Erkrankungen und Darmkrebs werden dabei in Kauf genommen. Die Gesamtemissionen der weltweiten Viehwirtschaft betragen 7,1 Gigatonnen CO_2_-Äquivalente pro Jahr, was 14,5 % der gesamten anthropogenen THG-Emissionen entspricht, die Rindertierzucht macht mit 65 % einen Großteil der Emissionen der Tierhaltung aus.

In Hinblick auf fundamentale menschliche Bedürfnisse ist aber auch die Abhängigkeit vom motorisierten (Individual‑)Verkehr als überaus kritisch zu sehen: Neben dem daraus resultierenden Beitrag zu Treibhausgasen resultiert daraus auch mangelnde körperliche Mobilität (Radfahren, Zu-Fuß-Gehen), die wiederum Übergewicht und Adipositas, insbesondere auch bei Kindern, fördert – mit allen gesundheitlichen Folgeschäden.

Die *zweite ethische Herausforderung* besteht darin, dass die gegenwärtigen THG-Emissionen tiefgreifende Auswirkungen auf die kommenden Generationen haben werden. Zwar ist im ärztlichen Gelöbnis keine explizite Erwähnung einer Zukunftsgerichtetheit zu finden, doch implizit scheint diese sehr wohl enthalten, etwa wenn gelobt wird, sich für die Verbesserung der Gesundheitsversorgung einzusetzen. Demzufolge scheinen auch Auswirkungen, welche die Gesundheit von zukünftigem Leben betreffen, durchaus relevant für die ethische Sichtweise der Ärzteschaft. THG wie Kohlenstoffdioxid, Lachgas, Methan und fluorierte Kohlenwasserstoffe wirken erst zeitverzögert, verbleiben in der Regel lange in der Atmosphäre und tragen über viele Jahrhunderte zu negativen Klimafolgen bei [19]. Die zukünftigen negativen Auswirkungen des Klimawandels werden zukünftig wesentlich schwerwiegender und überdies kumulativ zu Tage treten. Erschwerend kommt hinzu, dass die durch die Klimakrise ausgelösten Ereignisse teilweise unumkehrbar sind, z. B. das Abschmelzen des Grönlandeises, und sich gegenseitig im Sinne einer positiven Rückkoppelung verstärken. Nachfolgende Generationen werden, anders als bei anderen Krisen, wohl gar nicht mehr die Chance haben, daran maßgeblich etwas zu ändern. Besonders hervorzuheben ist hier ein wegweisendes Urteil des deutschen Höchstgerichts: Es sah in dem nicht gesetzten bzw. unzureichenden Handeln der Politik eine unfaire Lastverschiebung auf zukünftige Generationen und leitete dies aus mehreren Grundrechten ab [20].

Die nächsten beiden genannten ethischen Herausforderungen stehen in Verbindung mit dem Gelöbnis, dass Faktoren wie Alter, Geschlecht, soziale Stellung oder dergleichen ärztliche Pflichten nicht behindern dürfen. In einer Fortführung und Erweiterung dieses Gedankens erscheint es angebracht, auch präventiv gegen jene Aspekte vorzugehen, welche die medizinische Schlechterstellung von Angehörigen der genannten Gruppen begünstigen – und hier zählen der Klimawandel und seine Folgen jedenfalls dazu. Entsprechend betrifft die *dritte ethische Herausforderung* die globale Natur des Klimawandels. Einmal emittiert, können Treibhausgasemissionen überall auf dem Planeten zu klimatisch bedingten Folgen führen, unabhängig von ihrer Quelle [21]. Eine Ursache für den steten Temperaturanstieg ist die zunehmende Ausbeutung und Verschmutzung öffentlicher Ressourcen, die als *tragedy of the commons *[22] beschrieben und einer wachsenden menschlichen Bevölkerung zugeschrieben wurde (und von manchen nach wie vor wird). Besonders akut bis mittelfristig sind die vulnerabelsten Bevölkerungsgruppen und Länder genau diejenigen, die in der Vergangenheit am wenigsten THG emittiert haben und deren Emissionsniveau weiterhin vergleichsweise sehr niedrig ist.

Betrachtet man die Zeit seit Beginn der Industrialisierung, also etwa ab 1750, so haben die USA und Europa zusammen etwa die Hälfte der globalen CO_2_-Emissionen verursacht, während China bei rund 13 % liegt, Afrika und Südamerika machen zusammen hingegen etwa 6 % aus [23]. Dies zeigt ein völliges Ungleichgewicht auf und wirft einen bemerkenswert dunklen Schatten auf die Bemühungen um eine globale, respektvolle Zusammenarbeit. Die Nobelpreisträgerin Elinor Ostrom konnte bereits in den 1990er-Jahren zeigen, dass die Existenz bestimmter (gemeinsam verwalteter) Eigentumsrechte für eine gute Verwaltung von Gemeingütern notwendig ist [24, 25]. Auch die Tatsache, dass internationale Konzerne staatliches Recht immer wieder umgehen und ihre Produkte und Dienstleistungen z. B. in der EU verkaufen, aber ihre Emissionen „auslagern“, würde eine Governance of Commons auf internationaler Ebene unbedingt nötig machen.

Auf gesellschaftlicher Ebene ist erwähnenswert, dass soziale Gruppen mit dem höchsten Bildungsgrad, den höchsten Einkommenslagen und dem höchsten Umweltbewusstsein die meisten Ressourcen verbrauchen [26]. Sozioökonomisch schlechter gestellte Gruppen verfügen zwar über weniger Umweltbewusstsein, jedoch ist ihr ökologischer Fußabdruck um vieles kleiner, da sie sich ein ressourcenintensives Leben schlicht nicht leisten können.

Die offene Frage, wie man soziales und wirtschaftliches Wachstum in hiesigen Wirtschaftssystemen in Anbetracht dieser Tatsachen in Zukunft regeln soll, zeugt von den großen gesellschaftlichen Anstrengungen inmitten der Klimakrise.

Die *vierte ethische Herausforderung *ist eng mit dem vorhergehenden Punkt verknüpft: Die Klimakrise verstärkt bestehende geschlechtsspezifische bzw. vergeschlechtlichte Ungerechtigkeiten sowie auch die weitere Marginalisierung von Minderheiten [27]. Aus ethischer Sicht ist dies insofern ein Problem, da hinreichend bekannt ist, dass eine existentielle Schlechterstellung mit Negativfolgen für die psychische und physische Gesundheit verknüpft ist. Neben den negativen Auswirkungen chronischer Stressoren wirken sich viele Ungleichheiten auch auf grundlegende Faktoren der Lebenserhaltung wie z. B. Lebensmittelsicherheit aus. Somit ist eine Bekämpfung dieser Ungerechtigkeiten auch aus medizinisch-präventiver Sicht eine wesentliche Maßnahme. Als Beispiel sei die Gruppe der Frauen herausgegriffen: Diese hängen bei der Nahrungsmittelversorgung deutlich stärker von Subsistenzlandwirtschaft ab. Des Weiteren besitzen Frauen weniger Grund und Boden und haben weniger Zugang zu Land und daher weniger Möglichkeiten, Nahrungsmittel eigenständig zu produzieren. Wenn Lebensmittelpreise steigen, essen Frauen in ärmeren Familien oft weniger im Vergleich zu anderen Familienmitgliedern [28]. Auf der anderen Seite tragen Frauen weniger zu den CO_2_-Emissionen bei als Männer. So zeigt eine rezente schwedische Studie, dass auch im geografischen Norden Männer 16 % mehr Klima-Emissionen als Frauen verursachen (10 t CO_2_/Kopf/Jahr vs. 8,5 t CO_2_/Kopf/Jahr) [29]. Die Klimakrise schadet der Gesundheit von ungeborenem Leben, Säuglingen und Kleinkindern weltweit. So werden Hitzeperioden etwa mit Frühgeburten, rascherer Gewichtszunahme und vermehrten Krankenhauseinweisungen von Kindern in Verbindung gebracht. Ein Mechanismus für die rasche Gewichtszunahme bei Säuglingen besteht möglicherweise darin, dass zur Aufrechterhaltung der Körpertemperatur weniger Fett verbrannt werden muss, wenn die Umgebungstemperatur erhöht ist [30].

In der Klimakrise haben bekanntlich auch Waldbrände aufgrund zunehmender Trockenheit und Dürre weltweit stark zugenommen. Eine kalifornische Studie hat kürzlich einen signifikanten Zusammenhang zwischen der Exposition gegenüber Waldbränden 30 Tage vor einer Schwangerschaft sowie während des ersten Trimesters und der Entwicklung einer fötalen Gastroschisis dargestellt [31].

In einer rezenten Ausgabe von *Paediatric and Perinatal Epidemiology* [32] zu den nunmehr massiven klimatischen Auswirkungen zeigen zwei große Studien aus Australien [33] und den USA [34] den Zusammenhang zwischen Hitzewellen und dem gehäuften Auftreten spontaner Frühgeburten. Ein systematischer Review US-amerikanischer Daten (57 inkludierte Studien mit fast 33 Mio. Geburten) fand einen signifikanten Zusammenhang zwischen Hitze, Ozon oder Feinststaub (PM_2,5_) und einem erhöhten Risiko für Frühgeburten, niedriges Geburtsgewicht und Totgeburten [35].

Die *fünfte ethische Herausforderung* betrifft die Frage, inwiefern ethische Anliegen der Ärzteschaft auch das zugrundeliegende globale Gesellschafts- und Ökosystem mitbedenken sollen, wobei unsere theoretischen Werkzeuge in vielen der relevanten Bereiche noch nicht ausreichend entwickelt sind. Dazu zählen etwa internationale Gerechtigkeit, Gerechtigkeit zwischen den Generationen und Umgang mit wissenschaftlicher Unsicherheit. So wirft der Klimawandel, obwohl generell darüber kein Zweifel mehr besteht, vereinzelt auch Fragen über den Wert nicht-menschlicher Natur auf: Ob wir Verpflichtungen haben, Tiere, Pflanzen, einzigartige Orte oder die Natur bzw. Ökosysteme als Ganzes zu schützen, und welche Form solche Verpflichtungen annehmen, wenn wir dies tun [36]. Darüber hinaus setzen die wissenschaftliche Unsicherheit und das Potenzial für katastrophale Folgen den ökonomischen Standardansatz für Umweltprobleme unter Druck. Schon lange wird ein Ansatz im Sinne der Prävention und Vorsorge als Alternative angesehen [37]. Um den Schaden für die Betroffenen durch THG-Emissionen möglichst gering zu halten, wäre auch eine andere Herangehensweise hinsichtlich der Folgen prognostizierter Klimaveränderungen nach dem ärztlichen Ethos „primum nihil nocere“ wünschenswert, wie er von der hippokratischen Tradition ins Zentrum von moralisch gefordertem Handeln gestellt wird. Vollständig lautet das Zitat: „Primum non nocere, secundum cavere, tertium sanare.“ („Erstens nicht schaden, zweitens vorsichtig sein, drittens heilen“ [38]). Dieses Zitat stammt von Scribonius Largus, Arzt am Hof von Kaiser Tiberius Claudius.

## Fazit und Zusammenfassung

Die Klimakrise bedroht die elementaren natürlichen Lebensgrundlagen, von denen wir in praktisch allen Lebensbereichen abhängig sind [39]. Es handelt sich um ein komplexes globales Phänomen mit zahlreichen gravierenden Folgen für die Menschheit [40]. Die CO_2_-Konzentration in der Atmosphäre ist gegenwärtig höher als 418 ppm [41], die höchste seit mindestens drei Millionen Jahren [42] und ist eindeutig auf weiterhin steigende, anthropogene Emissionen zurückzuführen. Dies bedingt den seit Ende des 19. Jahrhunderts beobachteten [40, 43] und in den letzten Jahrzehnten stark zunehmenden Temperaturanstieg – im globalen Mittel um etwas mehr als 1 °C [44].

Die Klimaerhitzung hat vielfältige messbare sowie bereits beobachtbare gesundheitsrelevante Konsequenzen wie vermehrte Extremwetterereignisse [45], Dürre- und Flutkatastrophen [46], Waldbrände [47], Verlust von Biodiversität [48] sowie Ausbreitung von Infektionskrankheiten. Darüber hinaus treten hitzebedingte Gesundheitsprobleme wie Erschöpfung, Hitzschlag und Herzinfarkt auf [49, 50]. Diese sind die Folge anhaltender Feuchttemperatur (wet-bulb temperature) von mehr als 35 °C, eine Schwelle, bei der der menschliche Organismus nicht mehr in der Lage ist, die Haut ausreichend zu kühlen [51]. Weitere Folgen sind Zerstörung von Infrastruktur und Energieversorgung, beeinträchtigte Nahrungsproduktion mit Konflikten um Verknappung von Wasser und Versorgung mit Nahrungsmitteln, was wiederum zur Beeinträchtigung physischer und psychischer Gesundheit sowie möglichen Unruhen führen kann [52, 53]. All diese unmittelbaren und bekannten Negativfolgen auf die menschliche Gesundheit stellen für eine Ärzteschaft, die gelobt hat, dass Gesundheit und Wohlergehen ihrer Patienten und Patientinnen oberstes Gebot sei, eine ethische Herausforderung dar – zumindest, sofern sie nicht durch eigenes Verhalten versucht, die adversen Gesundheitsfolgen möglichst zu minimieren.

Darüber hinaus zeigen sich auch weitere mittelbare Folgen auf die Gesundheit, da der Klimawandel das Potenzial hat, bestehende (soziale) Spannungen zu verschärfen oder sogar neue hervorzurufen. Er kann ein Katalysator für gewaltsame Konflikte und eine Bedrohung für die internationale Sicherheit sein [54, 55], mit der möglichen Konsequenz weitreichender Migrationsbewegungen von Menschen – sowohl intranational als auch über bestehende nationale Grenzen hinweg [56–58].

Der Weltklimabeirat hat schon vor drei Jahrzehnten auf die massiven negativen Folgen des Nichthandelns hingewiesen [44]. Trotz der internationalen Anerkennung des ökologischen Gleichgewichts als Menschenrecht ist die Menschenwürde durch die Klimakrise zunehmend bedroht. Sie bringt große ethische Herausforderungen mit sich, bei denen es besonders um die Verantwortung gegenüber verletzlicheren Gruppen bzw. insgesamt allen Lebewesen in der Biosphäre geht. Ein wesentlicher Teil der ethischen Herausforderung der Gegenwart besteht im Hinterfragen des oft selbstverständlich vorausgesetzten exklusiven Anthropozentrismus. Unserer Ansicht nach wäre es von großer Bedeutung, den Menschen mit den anderen Lebewesen der Ökosphäre endlich als ein untrennbares, voneinander abhängiges Ganzes zu betrachten. Natur und Mensch koexistieren, wir sind Teil der uns umgebenden Ökosysteme und stehen nicht darüber – der Respekt gegenüber der Natur mit ihren für uns lebensnotwendigen Funktionen sollte derselbe sein wie der Respekt gegenüber anderen Menschen.

Eine Auseinandersetzung mit den Herausforderungen der Klimakrise auch aus ethischer Sicht wie etwa durch die österreichische Bioethikkommission ist daher höchst sinnvoll. Einerseits um die ethische Dimension des Nichthandelns in Bezug auf die weiter oben dargestellten drängenden Herausforderungen herauszuarbeiten, andererseits um die Durchsetzung entsprechender konkreter Maßnahmen zur Eindämmung der fatalen Auswirkungen auf derzeitige und künftige Generationen voranzutreiben.
